# Maternal factors associated with early childhood caries among 3–5-year-old children with low socio-economic status in Trishal, Bangladesh

**DOI:** 10.3389/froh.2023.1244359

**Published:** 2023-10-24

**Authors:** Farzana Haque, Morenike Oluwatoyin Folayan, Jorma Ilmari Virtanen

**Affiliations:** ^1^ Department of Clinical Dentistry, Faculty of Medicine, University of Bergen, Bergen, Norway; ^2^ Department of Child Dental Health, Obafemi Awolowo University, Ile-Ife, Nigeria; ^3^ Institute of Dentistry, University of Turku, Turku, Finland

**Keywords:** attitude, children, early childhood caries, health behavior, knowledge, mothers

## Abstract

**Introduction:**

Early Childhood Caries (ECC) is more prevalent in nations where a larger portion of the population resides below the poverty line. This study aimed to evaluate the connections between maternal awareness, attitudes, practices related to oral health, and the occurrence of ECC among children aged 3–5 years in Bangladesh, a low–middle income country with high level of poverty.

**Methods:**

This cross–sectional study recruited mother–child pairs with a focus on children aged 3–5 years from low socioeconomic backgrounds in Trishal, Bangladesh. Data collected included maternal oral health knowledge, attitudes, and behaviors. Clinical examinations were conducted to check for dental plaque on the upper central incisors' labial surfaces. ECC was identified using the ICDAS II index criteria. Associations between maternal oral health knowledge, attitudes, behaviors, and children's plaque score and caries status were analyzed using multivariable logistic regression, and adjusting for confounding variables (child's age, gender, mother's age, education, and number of children).

**Results:**

Among 532 mother-child pairs, 491 (93.2%) mothers were unaware of the role of fluoride in preventing caries, while 516 (97%) recognized the importance of using fluoridated toothpaste during brushing. Additionally, 520 (97.7%) mothers reported not knowing how to brush their child's teeth, and 87 (16.4%) brushed their children's teeth twice daily. Visible dental plaque was observed in 420 (78.9%) children, and 321 (60.3%) had ECC. Higher plaque score increased the odds of ECC in the study population (AOR: 5.617, 95% CI: 3.511–8.987).

**Conclusions:**

Mothers of preschool children with low socioeconomic status were poorly aware of caries preventive behaviors and had suboptimal oral health practices for their children. The plaque score was the only oral health factor that seems to increase the risk for ECC among children with low socio-economic status in Bangladesh. It is imperative to prioritize support and interventions aimed at improving oral hygiene practices to reduce ECC risk in this population.

## Introduction

Early childhood caries (ECC) is a dental condition that affects children under the age of 6 (1). It is a widespread issue, impacting approximately 530 million children globally (2). ECC tends to be more prevalent in countries with higher levels of poverty (3, 4). Poverty-related factors often hinder mothers from providing adequate oral health care for their children, including lower maternal education, and suboptimal maternal knowledge, attitudes, and behaviors regarding children's oral health (5–7).

Bangladesh, a low-income country with a population of 169.4 million, has a significant proportion of its population living below the poverty line (8). The prevalent poverty and income inequality contribute to a higher incidence of ECC among children from lower socioeconomic backgrounds in Bangladesh. Multiple risk factors, such as low maternal education, inadequate nutrition, poor oral hygiene practices, limited access to dental care, and environmental factors, contribute to this problem (9).

In Bangladesh, there exists a gender-based literacy gap, with women having a literacy rate of 72.8%, lower than the 76.5% rate for men (10). Cultural norms and economic constraints often restrict women's access to education (11). This disparity has considerable repercussions on child health in Bangladesh. Educated women tend to have more decision-making power within their households and communities, better access to healthcare services, and improved maternal and child health outcomes (12, 13). As mothers play a pivotal role in childcare in Bangladesh, their healthcare-seeking behavior significantly influences their children's health (14). Hence, the lower literacy rate among Bangladeshi women not only limits their opportunities but also affects the overall health and well-being of their children.

While low female literacy in Bangladesh has been linked to increased child mortality, morbidity, malnutrition, and stunted growth (12), there is limited understanding of how maternal education specifically impacts children's oral health risks. A significant lack of awareness among parents about proper dental care for their children is observed in Bangladesh (6, 15). Notably, up to 40% of preschool children in Bangladesh suffer from ECC (6, 16). Yet, there is currently no available information about the connection between maternal knowledge, attitudes, behaviors, and the prevalence of ECC in this population.

This study is grounded in the theory of planned behavior (17), which emphasizes the role of intention in driving behavior. According to this theory, attitudes, subjective norms, and perceived behavioral control influence an individual's intention to engage in a particular behavior, ultimately shaping their behavior. By comprehending the factors affecting maternal knowledge and behaviors concerning oral health, healthcare providers can design tailored interventions to enhance oral health and reduce dental diseases among children in Bangladesh. Consequently, this study aims to assess the oral health knowledge, attitudes, and behaviors of mothers with children under 5 years old in Trishal, Bangladesh, and investigate their association with the prevalence of early childhood caries (ECC) among preschool children.

## Methods

This cross-sectional clinical study was conducted in Trishal, Bangladesh and involved mothers and their children under the age of 5. In conjunction with the clinical investigation, participating mothers were administered a survey. Our primary focus was to evaluate the oral health practices of mothers and the oral health condition of their children. The study, titled “Prevention of Early Childhood Caries among Bangladeshi Children” (MMC/IRB/2021/349), received ethical approval from the Institutional Review Board of Mymensingh Medical College. The research was carried out in accordance with the principles of the Declaration of Helsinki, ensuring voluntary and anonymous participation.

The study area is situated in the Mymensingh division, which comprises four districts, including the Mymensingh district. Within the Mymensingh district, there exists an upazila known as Trishal (https://www.mapsofindia.com/world-map/bangladesh). According to the 2022 Bangladesh census, the estimated population of Trishal is approximately 480,196 individuals. Trishal upazila is further subdivided into Trishal Municipality and 12 unions, covering a total area of 348.1 square kilometers.

The ISPP-JWATNO project, an abbreviation for Income Support Program for the Poorest, is a collaborative initiative between the Bangladeshi government and the World Bank. Its primary goal is to extend financial support to economically disadvantaged mothers and their children under the age of five residing in specific administrative regions, comprising 43 upazilas, within the Rangpur and Mymensingh divisions. This project incentivizes the utilization of services related to child nutrition and cognitive development through Conditional Cash Transfers. Furthermore, it aims to strengthen the capacity of local unions in providing social safety net services. The overarching objective is to increase maternal engagement with these services and combat the prevalent issue of high malnutrition rates in the targeted regions.

Our study was conducted in collaboration with the ISPP (Income Support Program for the Poorest) and specifically focused on beneficiaries from the Trishal union. Within the Trishal union, there are approximately 1,400 mothers participating in the ISPP. Among this group, we extended invitations to mothers with children aged 3–5 years (*n* = 723) to voluntarily participate in our research. Of the ISPP beneficiary mother-child pairs, 532 (73.5%) expressed their willingness to partake in the study. Prior to their involvement, parents/guardians were provided with information about the study's anonymous nature and its potential benefits for children at risk of developing ECC. Participation in the study was entirely voluntary, and all requested information was treated as confidential.

### Survey

The survey was conducted from March to April 2022 among mothers of children who regularly attended community clinics for various programs aimed at improving their children's nutrition and development. A local dentist (FH), trained for this purpose, collected the information through face-to-face interviews at the Trishal Upazila health complex, utilizing a validated questionnaire. The questionnaire covered topics such as oral hygiene practices, dietary habits, and maternal background, including age, educational level, and the number of children.

The data collection utilized a reliable questionnaire (18, 19), which was translated into Bangla and back translated by an independent bilingual translator. The survey was carried out by a proficient dentist (FH) fluent in the local language and familiar with local customs. The questionnaire consisted of four sections: the background section for gathering socio-economic information and three additional sections focusing on maternal knowledge, attitude, and behavior regarding their child's oral health. A pilot survey involving 20 mothers was conducted, and the questionnaire underwent three iterative processes for content validation. The Cronbach's alpha coefficient for the questionnaire was 0.822.

Data on confounding variables included the child's and mother's ages at their last birthdays, the child's sex at birth, maternal education level (categorized as basic, primary, secondary, or tertiary), and the number of children in the family.

Maternal oral health knowledge was assessed using nine questions that explored topics such as the use of fluoride, oral hygiene practices, and parental roles in their child's oral health. Response options were “Correct,” “Don't know,” and “Incorrect,” with scores of 1 for agreement, 0 for disagreement, and “Don't Know.” The sum of these scores constituted the final oral health knowledge score for each respondent.

The section on maternal attitude included nine statements related to parental opinions, intentions, and perceptions regarding their child's tooth brushing and daily consumption of sugary products. Responses were recorded on a 5-point Likert scale, ranging from “Strongly Agree” to “Strongly Disagree.” Scores ranged from 1 to 5, with 1 and 2 indicating agreement and 4 and 5 indicating disagreement. The sum of these scores represented the final oral health attitude score for each mother.

The behavior section comprised seven questions, including four related to the use of dental services, toothpaste usage, and the need for adult assistance during tooth brushing. Responses were either “Yes” (score 1) or “No” (score 0) for the first four questions. Tooth brushing frequency for both the mother and the child was assessed using options ranging from “Twice a day” to “Never.” Recommended brushing twice a day scored 1 and all other responses 0. Cleaning methods for the child's teeth were recorded as “Toothbrush,” “Toothpaste,” “Washcloth/Gauze,” or “Water.” Using toothbrush scored 1 and all other options 0. The sum of these scores represented the oral health behavior score for each mother.

For the multivariate analysis, the sum scores for knowledge, attitude, and behavior were dichotomized. Maternal knowledge was categorized as either poor (0–5) or good (6–9), attitude as poor (0–6) or good (7–9), and behavior as poor (0–3) or good (4–7). Plaque scores were categorized as 0 for no plaque and 1 for the presence of plaque on any tooth surface. ECC was categorized as 0 for no caries (ICDAS = 0) and 1 for the presence of caries (ICDAS = 1–6) in one or more teeth. Maternal age was categorized as <26 years and ≥26 years, and education level as low (basic/primary) or high (secondary/tertiary).

### Clinical examinations

Clinical examinations were conducted following the survey, with both the mother and the examiner (FH) positioned knee-to-knee. The examinations involved examining the child's lip to check for the presence of plaque and early signs of tooth decay using a headlamp, a WHO CPI probe, and a dental mirror. Prior to the study, the examiner received additional training from an experienced dentist who heads a university department of pediatrics (JV).

To determine the occurrence of dental caries, we utilized criteria defined by the American Academy of Pediatric Dentistry, considering any sign of dental caries on any tooth surface as indicative of ECC (1). The clinical examinations used the ICDAS II index criteria, ranging from 0 to 6. ECC was categorized into non-cavitated lesions (ICDAS 1–2) and advanced lesions (ICDAS 3–6). Visible dental plaque was assessed on the labial surfaces of the upper central incisors and recorded as “No visible plaque,” “Plaque present at gingival margin only,” or “Abundant dental plaque covering more than the gingival margin of the tooth” (20). A pilot examination for 20 children was conducted within a 2-week interval, and the intra-examiner kappa reliability score was 0.9 for ECC and 0.74 for oral hygiene status.

### Statistical analyses

Statistical analyses were conducted using IBM SPSS Statistics version 23.0. The *χ*^2^ test was employed to examine associations between background variables and maternal knowledge, attitude, and behavior scores. Bivariate analysis was used to explore associations between ECC occurrence and independent variables (maternal knowledge, attitude, behavior, and children's plaque scores). A multivariate regression model was constructed to identify factors related to ECC in 3–5-year-olds, while adjusting for confounding variables. Adjusted odds ratios (AOR), 95% confidence intervals (CI), and *p*-values were calculated, with statistical significance set at 0.05.

## Results

Table 1 shows that the average age of the mothers was 25.9 years (with a standard deviation of 4.4), and the majority, 300 (56.6%), were 24 years old or younger. Among the mothers, 413 (77.6%) had two or more children, 69 (13.0%) had attained tertiary education, and 438 (82.3%) reported brushing their teeth once a day. The children had an average age of 45.5 months (with a standard deviation of 8.1), and the majority, 302 (56.8%), were under 48 months old. Among the children, 298 (56.0%) were boys, and 146 (27.4%) were first-born. In terms of dental conditions, 321 (60.3%) of the children had ECC (ICDAS 1–6), with 159 (29.9%) having non-cavitated lesions (ICDAS 1–3). Marginal plaque was observed in 224 (42.1%) of the children, while 196 (36.8%) had plaque in other areas.

**Table 1:** Basic characteristics of mothers and children 3–5 years old with low socioeconomic status resident in Trishal, Bangladesh (*N* = 532).

Characteristics	Total *n* (%)	Values	*n* (%)
Age of mother (years)	532 (100)		
		17–24	300 (56.6)
		>25	232 (43.4)
Mother's education	532 (100)		
		Basic	35 (6.6)
		Primary	265 (49.8)
		Secondary	163 (30.6)
		Tertiary	69 (13.0)
Nr of children	532 (100)		
		1	146 (27.4)
		2	267 (50.2)
		>2	119 (22.3)
Age of child (months)	532 (100)		
		36–47	302 (56.8)
		48–59	230 (43.2)
Sex	532 (100)		
		Boy	298 (56.0)
		Girl	234 (44.0)

Table 2 shows that out of the mothers, 490 (92.1%) recognized the significance of the primary teeth, while 517 (97%) were aware of the importance of cleaning children's teeth after birth and brushing them twice a day once the first tooth erupts. However, 491 (93.2%) of the mothers lacked awareness regarding the role of fluoride in preventing tooth decay, and 516 (97%) were unaware of the importance of using fluoridated toothpaste during tooth brushing. Additionally, 374 (70.3%) were uninformed about the link between soft drinks and dental caries, and 511 (96%) were unaware of the necessity for regular dental check-ups.

**Table 2:** Distribution of oral health knowledge status of mothers of children 3–5 years old with low socioeconomic status resident in Trishal, Bangladesh (*N* = 532).

Variable	Correct	Don’t know	Incorrect	Total
	*n* (%)	*n* (%)	*n* (%)	*n* (%)
Baby teeth are as important as the adult teeth	490 (92.1)	9 (1.7)	33 (6.2)	532 (100)
Tooth cleaning should start after birth	517 (97.2)	2 (0.4)	13 (2.4)	
Tooth brushing twice a day should start when the first tooth erupts	519 (97.6)	5 (0.9)	8 (1.5)	
Fluoride prevents decay	16 (3.0)	516 (97.0)	0	
It is best to use toothpaste with fluoride when brushing a child's teeth	36 (8.8)	491 (92.3)	5 (0.9)	
Soft drinks can cause tooth decay	158 (29.7)	356 (66.9)	18 (3.4)	
There's no need to go to the dentist unless children have problem with their teeth	21 (3.9)	23 (4.3)	488 (91.7)	
Mother should avoid sharing spoon	525 (98.7)	1 (0.2)	6 (1.1)	
Parents checking their child's teeth every month for changes or spots	518 (97.4)	14 (2.6)	0 (0.0)	

**Figure 1:**
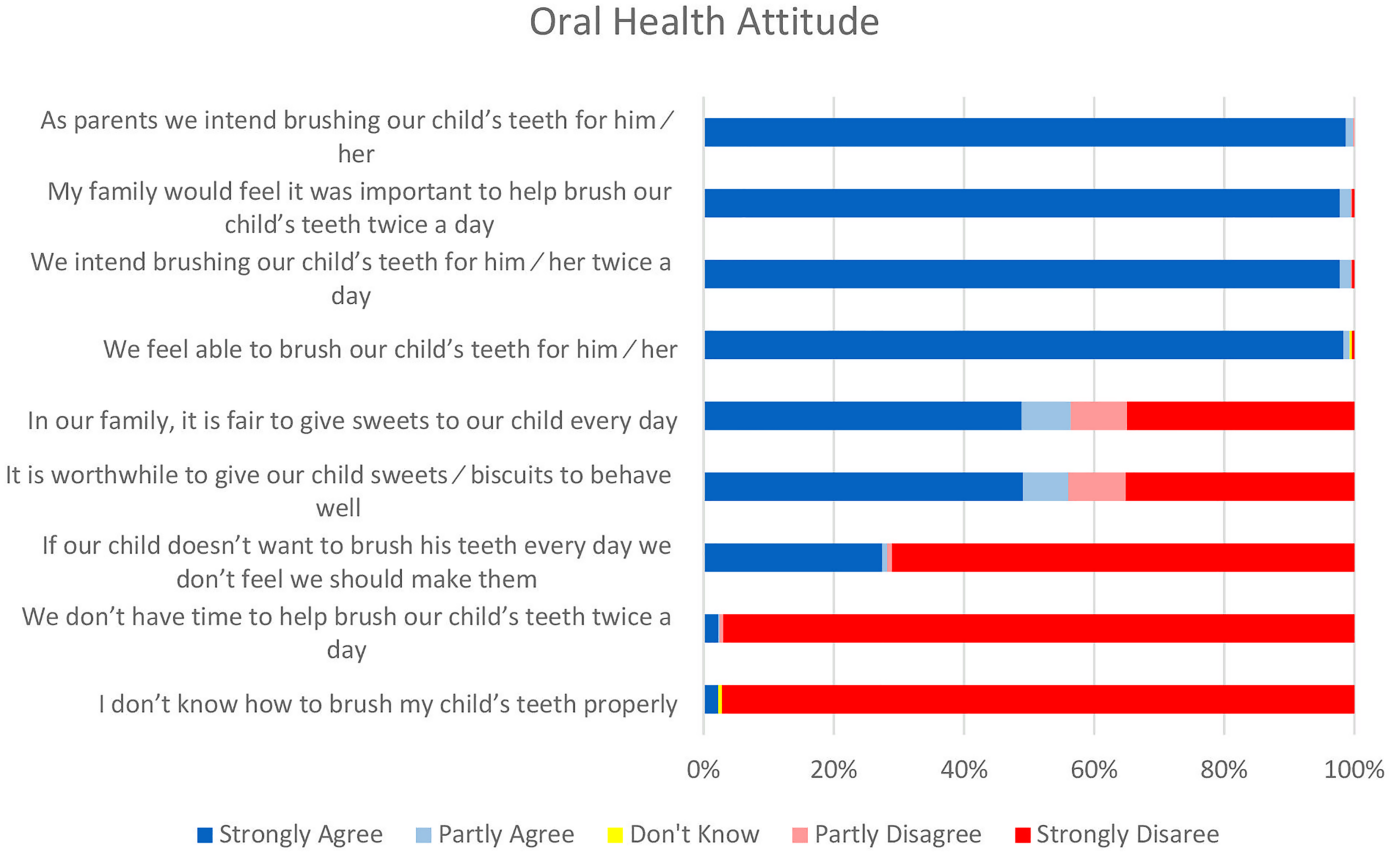
Attitude towards oral health among mothers of 3–5-year-old children with low socioeconomic status resident in Trishal, Bangladesh (*N* = 532).

Figure 1 illustrates the maternal attitudes regarding their children's oral health. Nearly all the mothers (99.8%) expressed their intention to brush their child's teeth twice daily. However, 520 (97.7%) admitted to not knowing the proper technique for brushing their child's teeth and cited a lack of time as a hindrance to brushing. Moreover, 231 (43.4%) believed it was acceptable to provide their child with sweets daily, and 233 (43.8%) considered it a rewarding practice to offer sweets and biscuits as a means of encouraging good behavior. Additionally, 382 (71.8%) of the mothers held the belief that if their child resisted tooth brushing, they should not insist on enforcing the practice.

Tables 3, 4 shows that 512 (96.2%) of the children had never undergone a dental check-up, and 515 (96.8%) had never visited a dentist. Moreover, 84 (15.8%) mothers assisted their children during the brushing process, 244 (45.9%) children brushed their teeth without toothpaste, 87 (16.4%) children had their teeth brushed twice daily, and 94 (17.7%) mothers brushed their own teeth twice a day. The educational level of the mothers exhibited a significant association with their oral health knowledge and behavior (*p* < 0.05). The number of children within the family was linked to mothers' knowledge (*p* = 0.005) and behavior (*p* = 0.04), while the child's age was associated with mothers' behavior (*p* = 0.005).

**Table 3:** Distribution of responses on oral health behavior status of mothers of children 3–5 years old with low socioeconomic status resident in Trishal, Bangladesh (*N* = 532).

Variable	Yes	No	Total
	*n* (%)	*n* (%)	*n* (%)
During the past year, has your child been to the dentist or dental clinic for a routine check-up or cleaning? (yes)	17 (3.2)	515 (96.8)	532 (100)
Has your child ever had his/her teeth checked by a dentist or other care provider? (yes)	20 (3.8)	512 (96.2)	532 (100)
Do you or another adult help your child brush his or her teeth?	84 (15.8)	448 (84.2)	532 (100)
When your child's teeth are brushed, is toothpaste usually used? (yes)	288 (54.1)	244 (45.9)	532 (100)

**Table 4:** Distribution of responses on tooth brushing habits of mothers and children 3–5 years old with low socioeconomic status resident in Trishal, Bangladesh (*N* = 532).

Variable		*n* (%)	Total *n* (%)
How often do you brush your own teeth?	Twice a day	94 (17.7)	532 (100)
	Once a day	438 (82.3)	
	Sometimes	0	
	Never	0	
How often are your child's teeth brushed?	Twice a day	87 (16.4)	
	Once a day	437 (82.1)	
	Sometimes	7 (1.3)	
	Never	1 (0.2)	
How do you clean your child's teeth?	Toothbrush	478 (89.8)	
	Toothpaste	37 (7.0)	
	Washed cloth/gauge	3 (0.6)	
	Water	14 (2.6)	

Table 5 provides the outcomes of the multivariate logistic regression analysis concerning the risk factors associated with ECC. In this study population, the presence of a plaque score emerged as the sole risk factor for ECC. Children with plaque had nearly six times higher odds of experiencing ECC compared to those without plaque (Adjusted Odds Ratio = 5.617, 95% CI: 3.511–8.987).

**Table 5:** Factors related to ECC of the 3–5-year-olds with low socioeconomic status in Trishal, Bangladesh (*N* = 532) explained by logistic regression model.

Variables	Total *N* = 532 *n* (%)	ECC		AOR; 95% CI; *p* value
		No Caries *N* = 211 (39.7%) *n* (%)	Caries present *N* = 321 (60.3%) *n* (%)	
Knowledge score				
Poor knowledge	369 (69.4)	134 (36.3)	235 (63.7)	0.702; 0.467–1.057; 0.090
Good knowledge	163 (30.6)	77 (47.2)	86 (52.8)	
Behavior score				
Poor Behavior	452 (85)	171 (37.8)	281 (62.2)	0.680; 0.403–1.149; 0.149
Good Behavior	80 (15)	40 (50)	40 (50)	
Plaque score				
No plaque	112 (21.1)	80 (71.4)	32 (31.2)	5.617; 3.511–8.987; <0.001
Plaque present	420 (78.9)	131 (28.6)	289 (58.8)	

Adjusted for: child's age, sex at birth, mother's age, educational status and number of children.

Omnibus test of model coefficients *χ*^2^ = 71.615; *p* < 0.001.

Nagelkerke R2 0.17.

## Discussion

Early Childhood Caries (ECC) is a prevalent issue in both developed and developing countries, and it was the central focus of this cross-sectional study conducted in Trishal, Bangladesh. The primary objective of the study was to pinpoint risk factors for ECC among children aged 3–5 years who came from low socio-economic backgrounds. The study uncovered a significant prevalence of ECC (60.3%) in this group of children, shedding light on the plaque score as a potential key risk factor associated with ECC.

One of the strengths of this study lies in its comprehensive data collection, involving surveys and clinical assessments of both mothers and children. The use of the ICDAS criteria enabled the identification of non-cavitated lesions, allowing for a thorough evaluation of caries experience in the study population. Furthermore, the study employed a multivariate regression model that incorporated various potential risk factors, enhancing its ability to predict ECC among the participants. Lastly, the study's representativeness was a major strength, as it drew from a sample that was a fair reflection of the target population.

However, there were a few limitations to the study. However, certain limitations must be acknowledged. Firstly, the study's sample was not entirely representative of all children in Bangladesh, as it exclusively recruited participants from a specific area within Trishal. Consequently, the applicability of the findings to other regions within the city or to children across Bangladesh may be somewhat limited. Additionally, the study predominantly featured individuals from lower socio-economic backgrounds, resulting in an underrepresentation of children from higher socio-economic strata. Nonetheless, despite this limitation, the study's findings bear significance, given their focus on identifying potential risk factors for ECC within a population characterized by low socio-economic status, a group that has been identified as being at high risk for ECC. Furthermore, the study did not collect data on factors such as malnutrition, developmental defects of teeth, systemic health conditions, and birth weight, which could have contributed to a more comprehensive understanding of the situation.

Previous research has consistently shown that children with low socio-economic status face a heightened susceptibility to ECC. A myriad of factors contributes to this increased risk, including financial constraints (3), restricted access to dental services (21), limited knowledge of effective oral hygiene practices (9), and insufficient resources for proper dental care (22). Furthermore, unhealthy dietary habits, such as the consumption of sugary snacks and beverages (23), exacerbate the prevalence of dental caries within low-income communities.

Our study aligns with the findings of prior research in several ways. Firstly, like earlier investigations (24–26), we identified plaque as a significant risk factor for ECC. Plaque plays a pivotal role in ECC development by generating acid as it metabolizes sugars and carbohydrates in the oral cavity. Over time, this acid gradually erodes tooth enamel, giving rise to cavities and other dental complications (27). Poor oral hygiene practices, such as irregular tooth brushing and flossing, contribute to plaque buildup, further elevating the risk of ECC (20). The low proportion of children who brush their teeth twice a day and utilize toothpaste during brushing substantially amplifies the ECC risk in this particular group of children. Consequently, there's an urgent need for strategic interventions aimed at identifying and mitigating the risk factors associated with suboptimal oral hygiene practices, especially within this population.

Secondly, our study findings indicate that, in contrast to some previous studies linking maternal oral health knowledge to ECC risk (28–30), this might not be a primary risk factor for ECC in this context. Unlike findings from other studies (28–30), our research did not demonstrate such a direct association. However, it's essential to interpret this result cautiously. One plausible explanation for this lack of association could be the high homogeneity within our study participants. Most of our study population belonged to low socio-economic strata, with over two-thirds possessing limited knowledge of oral health and a limited understanding of ECC risk factors. This lack of awareness may also explain the absence of a clear link between behavior and ECC experience, despite the presence of inadequate preventive measures against dental caries. Previous studies have indicated that poor maternal behavior can contribute to a higher prevalence of ECC (7, 28, 31).

Overall, our findings underscore the potential significance of enhancing oral hygiene practices as a crucial intervention to curb ECC within a high-risk population of children in Bangladesh. While the World Health Organization (WHO) offers general guidelines for ECC prevention and management (32), it's vital to formulate context-specific strategies tailored to the unique challenges of ECC prevention in this setting. Even though our study didn't uncover a statistically significant link between ECC and maternal knowledge of caries prevention measures, prior research has highlighted a robust connection between maternal knowledge and ECC risk through various pathways, including its impact on oral hygiene practices (20, 21). Maternal knowledge is just one of several factors that can influence behavior related to good oral hygiene practices for their children. In the short term, educating mothers on how to improve their children's oral hygiene through programs encompassing antenatal, natal, and postnatal care could yield positive outcomes (9, 33). Additionally, exploring other potential measures to reduce ECC risk in this population, with a strategic focus on improving plaque scores among preschool children, is essential. Currently, systematic public dental services for young children are lacking in Bangladesh. Although private services exist in larger cities, they remain inaccessible to most people, especially those from low socio-economic backgrounds, due to prohibitive costs.

In conclusion, the prevalence of ECC was high among preschool children in Trishal, Bangladesh. Mothers from lower socioeconomic backgrounds exhibited poor oral health knowledge and behaviors, with plaque score identified as the primary risk factor for ECC. The study highlights the importance of enhancing oral health knowledge and hygiene practices through maternal education as a crucial intervention to prevent ECC in Bangladesh. It is recommended to integrate oral health promotion and caries prevention into primary care services for mothers and their young children to improve overall oral health.

## Data Availability

The original contributions presented in the study are included in the article/Supplementary Material, further inquiries can be directed to the corresponding author.
